# Effects of a Western Diet on Colonic Dysbiosis, Bile Acid Dysmetabolism and Intestinal Inflammation in Clinically Healthy Dogs

**DOI:** 10.1111/jvim.70035

**Published:** 2025-03-20

**Authors:** Brandon Mason, Dipak Kumar Sahoo, Chelsea A. Iennarella‐Servantez, Aarti Kathrani, Shannon M. Morgan, Agnes Bourgois‐Mochel, Alex M. Bray, Vojtech Gabriel, Christopher Zdyrski, Jennifer M. Groeltz, Eric Cassmann, Mark R. Ackermann, Jan S. Suchodolski, Jonathan P. Mochel, Karin Allenspach, Albert E. Jergens

**Affiliations:** ^1^ Department of Veterinary Clinical Sciences, College of Veterinary Medicine Iowa State University Ames Iowa USA; ^2^ Department of Biomedical Sciences, College of Veterinary Medicine Iowa State University Ames Iowa USA; ^3^ Department of Clinical Science and Services Royal Veterinary College Hertfordshire UK; ^4^ Department of Pathology, College of Veterinary Medicine University of Georgia Athens Georgia USA; ^5^ National Animal Disease Center USDA, ARS Ames Iowa USA; ^6^ Gastrointestinal Laboratory, School of Veterinary Medicine and Biomedical Sciences Texas A&M University College Station Texas USA

**Keywords:** chronic inflammatory enteropathy, dysbiosis, fecal bile acids, high‐fat diet

## Abstract

**Background:**

Consumption of a high‐fat, high‐carbohydrate Western‐style diet (WD) associated with obesity and inflammation in humans has not been investigated in dogs.

**Aims:**

To determine the effects of WD on inflammatory indices, microbiome, and fecal bile acids (BAs) in dogs.

**Animals:**

Ten adult clinically healthy dogs.

**Methods:**

A dietary trial compared the effects of two home‐prepared diets: a high‐fiber, low‐fat control diet (CD) to a diet containing the macronutrient composition of WD (low‐fiber, high fat). Dietary treatments were given sequentially for three feeding periods, each lasting 1 month. Outcome measures included molecular/microbiologic testing of colonic biopsies, histopathology, inflammatory biomarkers, and quantification of fecal BA following each feeding period.

**Results:**

Cell markers of apoptosis (TUNEL‐positive cells: CD1, 0.36% ± 0.2%; WD, 0.79% ± 0.5%; CD2, 0.42% ± 0.3%; 95% CI) and inflammation (NF‐ĸB area: CD1, 8.09% ± 3.3%; WD, 11.58% ± 3.4%; CD2 7.25% ± 3.8%; 95% CI), as well as serum high‐sensitivity C‐reactive protein (CD1, 2.0 ± 0.4 ng/mL; WD, 2.76 ± 0.23 ng/mL; CD2, 2.29 ± 0.25 ng/mL; 95% CI), were increased (*p* < 0.05) in dogs fed WD versus CD. Other perturbations seen with WD ingestion included altered (*p* < 0.05) colonic mucosal bacteria (bacterial counts: CD1, 301.5 ± 188.5; WD, 769.8 ± 431.9; CD2, 542.1 ± 273.9; 95% CI) and increased (*p* < 0.05) fecal cholic acid (median and interquartile range/IQR: CD1, 9505 [2384–33 788] peak heights; WD, 34 131 [10 113–175 909] peak heights) and serum myeloperoxidase (CD1, 46.98 ± 16.6 ng/mL; WD, 82.93 ± 33.6 ng/mL; CD2, 63.52 ± 29.5 ng/mL; 95% CI).

**Conclusions and Clinical Importance:**

WD fed to clinically healthy dogs promotes colonic dysbiosis, altered fecal BA, and low‐grade inflammation independent of obesity.

**Abbreviations**
AMDR: Acceptable Macronutrient Distribution Ranges
BA: bile acid
CA: cholic acid
CD: control diet
CDCA: chenodeoxycholic acid
CIE: chronic inflammatory enteropathy
DCA: deoxycholic acid
fUBA: fecal unconjugated bile acid
HFD: high‐fat diets
hs‐CRP: high‐sensitivity C‐reactive protein
IBD: inflammatory bowel disease
IRE: immunosuppressant‐responsive enteropathy
LCA: lithocholic acid
MPO: myeloperoxidase
NAS: National Academies of Sciences
NF‐ĸB: nuclear factor ĸB
NHANES: National Health and Nutrition Examination Survey
UDCA: ursodeoxycholic acid
WD: Western‐style diet

## 1 Introduction

The Western‐style diet (WD) is thought to be an unhealthy diet, as it is characterized by a high daily intake of calorically rich, high‐fat, high‐carbohydrate ingredients that are increasingly ingested by modern society [1, 2]. This dietary pattern is associated with higher consumption of refined sugars, animal fat, processed (red) meats, high‐fructose sugar, and salt, with many ingredients being processed, refined, fried, and prepackaged. In addition, the typical WD intake is also associated with reduced consumption of fish, grass‐fed animal products, whole grains, fruits, vegetables, and nuts. Therefore, WD has a high likelihood of being deficient in fiber, minerals, vitamins, and beneficial antioxidants [3, 4, 5]. Prolonged WD ingestion has been linked to diverse pathophysiological conditions in people, including obesity, dyslipidemia, Type‐2 diabetes mellitus, inflammatory bowel disease (IBD), cancer, cardiovascular disease, and cognitive impairment [6, 7, 8, 9, 10, 11, 12].

While there is a lack of studies on the effect of the health of dogs on WD, high‐fat diets (HFD, containing meat or meat products but devoid of carbohydrates and fiber) have been investigated for their effect on the canine microbiome and metabolome. In one study of healthy adult beagles, short‐term feeding of increased amounts of dietary fat resulted in a significant shift in fecal abundance of bacteria related to fat digestion [13]. Two other studies have evaluated changes in the fecal microbiome and metabolome of dogs fed HFD versus commercial diets. In one study, dogs fed a bones and raw food (BARF) diet showed a significant difference in beta diversity of fecal bacteria with increased abundance of *Escherichia coli* and *Clostridium perfringens* as compared to conventionally fed dogs [14]. However, there were no significant differences in the fecal metabolome, including bile acid (BA) concentrations, between diet groups. Another study showed that a diet shift from commercial dry food to HFD, and vice versa, influenced fecal BA concentrations in healthy adult dogs [15]. Here, the fecal concentration of the secondary BAs (deoxycholic acid [DCA] and ursodeoxycholic acid [UDCA]) was significantly increased in HFD samples compared with the concentration in commercial diet samples.

The convergence of studies in different species implicating interactions between HFD, dysbiosis, and BA dysmetabolism has important considerations for the maintenance of mucosal homeostasis. HFD induces dysbiosis and promotes BA dysmetabolism and low‐grade intestinal inflammation in humans [1] and animal models [16] of gastrointestinal (GI) disease. Whether healthy dogs fed WD have a similar pathogenic scenario promoting intestinal inflammation has not been reported. This study aimed to determine the effects of a high‐fat, high‐carbohydrate, low‐fiber WD on intestinal inflammation (histopathology, tissue nuclear factor ĸB [NF‐ĸB] expression), oxidative stress (serum myeloperoxidase [MPO]), systemic inflammation/oxidative stress (serum high‐sensitivity C‐reactive protein [hs‐CRP], serum MPO), colonic mucosal bacteria (fluorescence in situ hybridization [FISH]), mucosal apoptosis (TUNEL assay), and select fecal BA concentrations in clinically healthy dogs.

## 2 Materials and Methods

The study protocol was reviewed and approved according to the guidelines of the Iowa State University (ISU) IACUC committee (approval: IACUC‐19‐337).

### 2.1 Animals

The study cohort was comprised of 10 (five neutered males, five neutered females) clinically healthy adult Beagles (body condition scores of 4–5 each, weight range [kg] 8.7–11.7, maintenance energy requirement [MER] range: 512–670 cal) maintained as a closed colony within Laboratory Animal Resources (LAR) at ISU. All dogs were judged to be healthy based on normal physical examination findings and the absence of abnormalities observed on complete blood count, biochemistry, urinalysis, and fecal examinations for nematode/protozoal parasites. Each dog was immunized with all recommended vaccines. Dogs were fed a commercial dry maintenance ration, which provided 100% of their daily MERs before trial enrollment.

### 2.2 Diets and Study Design

Two custom‐designed, home‐prepared diets were formulated by computer software for the dietary trial. The first diet consisted of a high‐fiber, low‐fat control diet (CD) and the second diet, a low‐fiber, high‐fat Western‐style diet (WD). Both diets were formulated by a board‐certified veterinary nutritionist to ensure they were adequate for long‐term feeding and met the recommended allowances and minimum requirements of the National Research Council (NRC) 2006 nutritional profiles for canine adult maintenance. In addition to meeting the NRC profiles for canine adult maintenance, both diets were formulated to also meet the desired dietary macronutrient profiles based on the National Academies of Sciences (NAS), Acceptable Macronutrient Distribution Ranges (AMDR), and National Health and Nutrition Examination Survey (NHANES) dietary intake data from 2017 to 2018, respectively for the CD and WD (Tables 1 and 2). Both diets comprised the same ingredients, but at different amounts to ensure the desired macronutrient profiles for the respective diets in Table 1 were met (Table 3).

**TABLE 1:** Macronutrient profile of desired formulation of CD1/CD2 and WD.

	ME (kcal)	Fat (g)	CHO (g)	Sugar (g)	Fiber (g)	Protein (g)
AMDR MF (CD1/CD2)	1000.00	27.80	137.50	51.50	14.30	50.00
NHANES (WD)	1000.00	40.80	118.30	51.40	8.40	40.00

*Note:* The macronutrient profile that was desired for the control diet (CD1/CD2) and WD, which was based on the AMDR for the CD and the NHANES (https://www.ars.usda.gov/ARSUserFiles/80400530/pdf/1112/tables_1‐40_2011‐2012.pdf) for the WD.

Abbreviations: AMDR, Acceptable Macronutrient Distribution Ranges; CD, control diet; CHO, carbohydrate; ME, metabolizable energy; MF, moderate fat; NHANES, National Health and Nutrition Examination Survey; WD, Western diet.

**TABLE 2:** Macronutrient profile of final formulation of CD1/CD2 and WD.

	FED (g)	ME	Fat (g)	CHO (g)	Sugar (g)	Fiber (g)	Protein (g)	Cost ($)
AMDR MF (CD1/CD2)	830.20	1000.00	28.22	136.47	50.69	14.01	50.93	0.00
NHANES (WD)	617.00	998.97	40.92	120.03	48.66	7.79	38.98	0.00

*Note:* The macronutrient profile that was achieved following the formulation of the CD (CD1/CD2) and WD, which was based on the AMDR for the CD and the NHANES (https://www.ars.usda.gov/ARSUserFiles/80400530/pdf/1112/tables_1‐40_2011‐2012.pdf) for the WD.

Abbreviations: AMDR, Acceptable Macronutrient Distribution Ranges; CD, control diet; CHO, carbohydrate; ME, metabolizable energy; MF, moderate fat; NHANES, National Health and Nutrition Examination Survey; WD, Western diet.

**TABLE 3:** Ingredients and amounts present in diet formulation of CD and WD.

Ingredient	CD1/CD2		WD	
	Grams per 992 kcal	Grams per 1000 kcal	Grams per 618 kcal	Grams per 1000 kcal
Ground beef (25% fat), raw amount, fed cooked	55.00	55.44	62.00	100.32
Fresh egg white, raw amount, fed cooked	275.00	277.22	77.00	124.60
Brown rice, long‐grain, cooked amount	150.00	151.21	62.00	100.32
White rice, long‐grain, cooked amount	52.00	52.42	62.00	100.32
Oatmeal, multigrain, prepared with water, cooked amount	225.00	226.81	74.00	119.74
Corn syrup, light	62.00	62.50	38.00	61.49
Corn oil	5.00	5.04	3.00	4.85
Lard	2.00	2.02	3.00	4.85
Almonds	8.00	8.06	5.00	8.09
Pure psyllium seed husk	8.00	8.06	2.00	3.24
Morton iodized salt	—	—	0.38	0.61
Balance IT canine supplement	18.75	18.90	—	—
Balance IT canine K supplement	—	—	9.00	14.56
Freeda calcium phosphate powder	—	—	1.40	2.27

*Note:* The ingredients and their respective amounts present in the control diet (CD1/CD2) and Western diet (WD). Due to the different amounts of base ingredients needed for the two diet categories to meet the desired macronutrient composition, different supplements were needed in each category (i.e., Balance IT canine supplement for CD and balance IT canine K supplement and freeda calcium phosphate powder for WD) to ensure the diets met the recommended allowances and minimum requirements of the National Research Council (NRC) 2006 nutritional profiles for canine adult maintenance.

**FIGURE 1:**
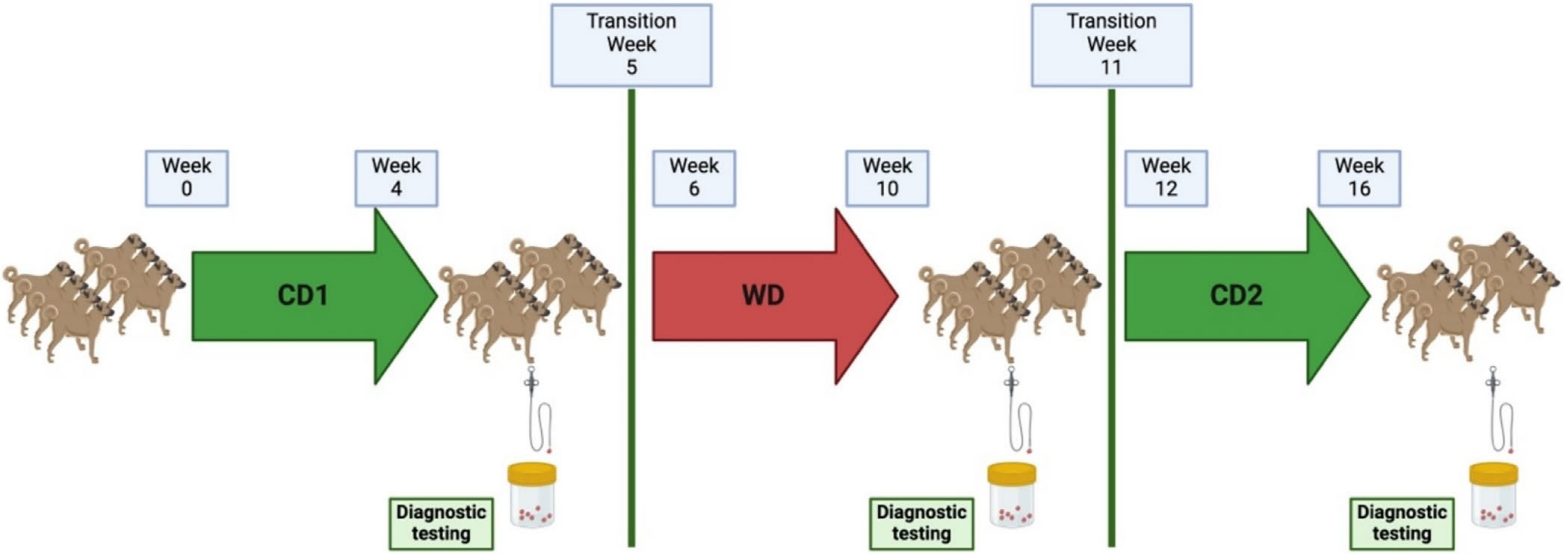
Study design of the dietary trial. Dietary treatments were provided sequentially for three feeding periods, each lasting 1 month in duration. There was a one‐week transition when moving from one feeding period to the next. Diagnostic testing was performed at the completion of each feeding period.

The home‐cooked diets were prepared once weekly for all dogs in one batch that was then divided into individual meals for each dog for seven consecutive days. Individual meals were placed in zip‐lock bags and stored at −20°C until used. The evening before being fed, each dog's individual diets were removed from the freezer and allowed to thaw in their plastic bags at room temperature before the scheduled feeding the next morning. This same procedure for diet preparation and storage was followed during each week of the feeding trial, albeit with different diets. Diets were fed isocalorically to each dog based on calculated metabolizable energy (ME) values. Dietary treatments were provided sequentially for three feeding periods, each lasting 1 month in duration, with a weeklong transition period between each change in diet. Diets were assigned based on an ABA study design with CD fed for Periods 1 and 3 and WD fed for Period 2 (Figure 1) [17].

Biological specimens (serum, feces) were obtained from all dogs at the completion of each feeding period and archived as CD1, CD2, and WD. Following an 18‐h period where food was withheld, colonoscopy with mucosal biopsy was performed in eight dogs at the completion of the CD1, WD, and CD2 periods. Dogs were prepared for GI endoscopy in a standard fashion using a commercial polyethylene glycol solution with electrolytes administered at 20 mL/kg PO in two doses [18]. Following colonic lavage, dogs were placed under general anesthesia and 12–15 endoscopic biopsy specimens were obtained from all colonic regions for histopathologic and microbiologic review.

### 2.3 Colonic Histopathology

Histopathologic examination of colonic biopsies was performed by a single pathologist (M.R.A.) blinded to each dog's diet and feeding period (e.g., CD1, WD, CD2). Mucosal biopsies were assessed for intestinal inflammation using modified WSAVA histopathologic guidelines, where morphologic and inflammatory features were graded independently and summed into a total histopathologic score [19]. Alcian blue staining was performed to identify the number of goblet cells in tissue sections. The percentage of Alcian blue staining was quantified using Halo software.

### 2.4 Biomarkers of Inflammation, Oxidative Stress, and Apoptosis

Local (immunohistochemistry [IHC] for colonic mucosal expression of NF‐ĸB) and systemic (serum hs‐CRP) biomarkers were evaluated in dogs at the completion of each feeding period. Immunohistochemical staining was performed using an NF‐ĸB p65 (D14E12) XP rabbit monoclonal antibody for protein detection at a dilution of 1:500 (Cell Signaling Technology, Danvers, MA). Automated staining from deparaffinization through counterstaining took place on Roche Diagnostic's DISCOVERY ULTRA IHC/ISH platform, with all subsequent reagents listed in this procedure being from Roche Diagnostics (Indianapolis, IN). Four‐micron canine colon tissue sections mounted on glass slides from paraffin‐embedded blocks were baked at 60°C before placement on the stainer. After online deparaffinization with EZ Prep Solution, heat retrieval at 100°C with ULTRA Cell Conditioning 1 was applied for 48 min. This was followed by endogenous peroxidase blocking with Inhibitor CM for 8 min. The primary antibody was next applied at 37°C with an incubation time of 60 min. A linking antibody (anti‐Rabbit HQ at 37°C) followed by an enzyme conjugate (anti‐HQ HRP at room temperature) were both applied for 12 min each. Detection and staining were completed using the DISCOVERY ChromoMap DAB Kit, followed by counterstaining with Hematoxylin I for 8 min and post‐counterstaining with Bluing Reagent for 4 min. Fiji (ImageJ) was employed for the purpose of performing deconvolution and subsequent semiquantitative analysis of IHC images [20].

hs‐CRP in serum was analyzed by sandwich ELISA according to the manufacturer's instructions (https://www.mybiosource.com/hs‐crp‐canine‐elisa‐kits/high‐sensitivity‐c‐reactive‐protein/734747; MyBioSource Inc., San Diego, CA, USA) [21]. For estimating MPO activity, quantitative sandwich ELISA was performed using canine MPO, MPO ELISA Kit (MyBioSource Inc., San Diego, CA) following the manufacturer's protocol.

Cell death in colon tissue sections was detected using the TUNEL Assay Kit‐HRP‐DAB (Abcam, Boston, MA), following the manufacturer's protocol. In brief, tissue slides were deparaffinized and nuclei were stripped of proteins by incubation with proteinase K for 20 min. After being treated with 3% hydrogen peroxide (H_2_O_2_) for 5 min and following the washing process, the slides were incubated with a TdT labeling reaction mix containing TdT enzyme in a humidified chamber at 37°C. The detection of tagged nucleotides was accomplished using streptavidin‐horseradish peroxidase (HRP) conjugate. Following the washing process, sections were stained using a solution of diaminobenzidine (DAB) and H_2_O_2_. The stained sections were then counterstained with methyl green, dehydrated, and subsequently mounted and imaged for analysis. The results are reported as the percentage of TUNEL‐positive cells out of the total number of lamina propria cells.

### 2.5 Fecal Unconjugated Bile Acids (fUBA)

The relative abundance of fecal unconjugated cholic acid (CA), chenodeoxycholic acid (CDCA), lithocholic acid (LCA), and DCA was measured in lyophilized feces using a dilution gas chromatography–mass spectrometry (GC‐MS) method (UC Davis Metabolomics Core Facility) as previously described in all samples collected [22]. Relative concentrations of primary (CA and CDCA) and secondary (LCA, DCA) fUBA were expressed as individual primary fUBA and secondary fUBA. Results were reported as peak heights of lyophilized feces comprising the measured fUBA pool.

### 2.6 Colonic Mucosal Bacteria

Formalin‐fixed, paraffin‐embedded colonic tissue sections (4 μ) were mounted on glass slides and evaluated for mucosal bacteria using FISH [21, 23, 24]. Tissue specimens were deparaffinized using an automated system by passage through xylene (3 × 10 min), 100% alcohol (2 × 5 min), 95% ethanol (5 min), and finally 70% ethanol (5 min). The slides were transported in deionized water to the DNA laboratory, where they were air‐dried before hybridization. FISH probes, 5′‐labeled with either Cy‐3 or FITC (Thermo Fisher Scientific, Rochester, USA) were reconstituted with nuclease‐free water and diluted to a working concentration of 5 ng/μL. Probe EUB338‐FITC was used for total bacterial counts. For other analyses, specific probes directed against Clostridium (EREC482) [25], Bacteroides (BAC303) [26], and Enterobacteriaceae (EBAC1790) [27] were labeled with Cy‐3 and applied simultaneously with the universal bacterial probe Eub338‐FITC. Specific probes were selected to identify bacterial groups relevant to the pathogenesis of intestinal inflammation in dogs [28, 29]. Tissue sections were immersed in 30 μL of DNA–probe mix in a hybridization chamber maintained at 54°C overnight (12 h). Washing was performed using a wash buffer (hybridization buffer without SDS); the slides were rinsed with sterile water, then allowed to air dry, and mounted with SlowFade Gold mounting media (Life Technologies, Carlsbad, CA, USA) and a 25 × 25–1 cover glass (Fisher Scientific, Pittsburgh, PA, USA).

Sections were examined on a Zeiss AxioImager Z.2 epifluorescence microscope (Dublin, CA, USA) and images were captured with a Zeiss MRM AxioCam camera (Dublin, CA, USA; www.zeiss.com; accessed on April 4, 2022). The number of colonic bacteria and their spatial distribution (free mucus, adherent mucus, surface epithelia, within mucosa) were quantified in ten 40× fields of two or more tissue sections using MetaMorph software. Results were expressed as mean bacteria per 40× field.

### 2.7 Statistics

Mucosal bacterial counts, percentage of TUNEL‐positive cells, MPO, and hs‐CRP activities data among different treatment periods were compared using one‐way repeated measures ANOVA followed by Tukey's multiple comparisons test by GraphPad Prism 9 (https://graphpad.com/). Minimal statistical significance was accepted at *p* < 0.05. The Shapiro–Wilk test was employed to assess the normality of the data. Statistical significance was determined at *p* < 0.05. Data that adhered to a normal distribution were presented as mean ± SD, while the data that did not follow a normal distribution were presented as median and range, and a nonparametric statistical test, Friedman test, was used for analyzing repeated measures data. Pearson's correlation coefficients were employed to evaluate the relationship between the effects of WD on inflammatory indices, oxidative damage, microbiome, and BA, with minimal statistical significance accepted at *p* < 0.05.

## 3 Results

**FIGURE 2:**
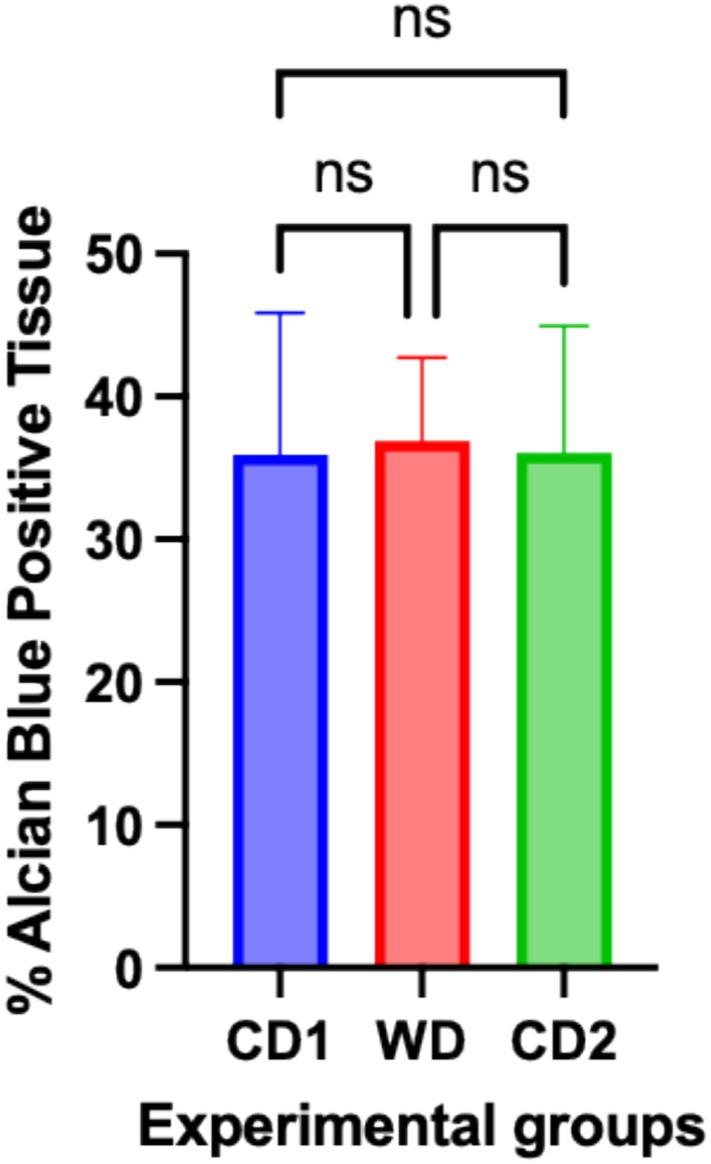
Percent Alcian blue staining in colonic mucosal biopsies of dogs fed CD or WD over the treatment schedule. The percentage of Alcian blue staining was quantified using Halo software. Data are presented as mean ± SD. CD1 = Control Diet Period 1; CD2 = Control Diet Period 2; ns = no significant difference; WD = Western diet.

Histopathologic inflammation was graded as absent to mild in colonic biopsies of dogs fed the control rations (CD1/CD2 feeding periods). Total histopathologic scores ranged from 0 to 1 and were characterized by increased lymphoplasmacytic mucosal infiltrates in some dogs. Dogs fed WD showed mild inflammatory lesions (total histopathologic score range, 1–2), with individual dogs having superficial mucosal edema and crypt distortion. There was no significant difference (*p* > 0.05) in the percentage of Alcian blue staining in dogs fed CD as compared to dogs fed WD (CD1, 35.94 ± 9.9; WD, 36.9 ± 5.8; CD2, 36.08 ± 8.8; 95% CI; Figure 2).

**FIGURE 3:**
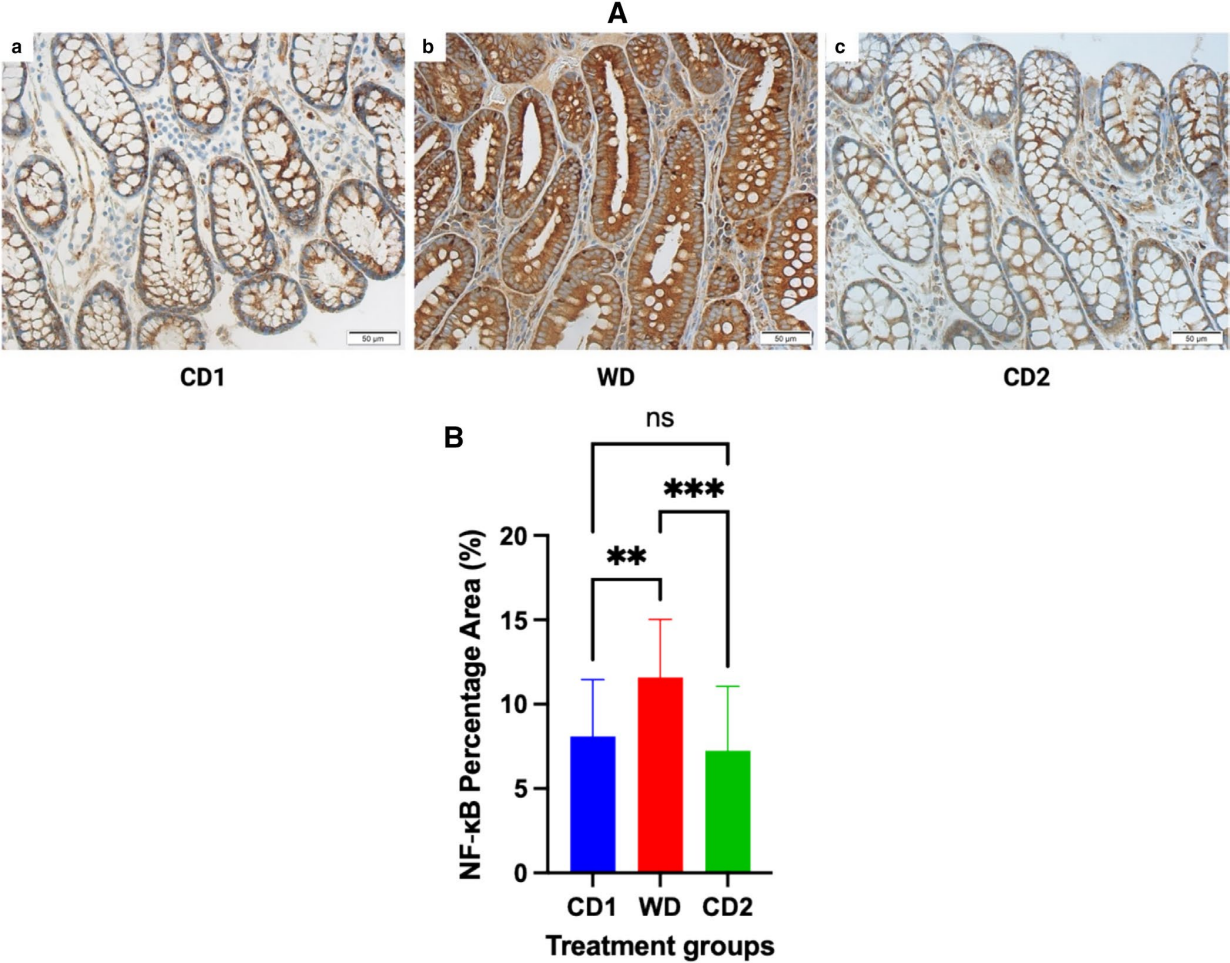
(A) Immunohistochemical analysis of NF‐ĸB expression/mucosal field in colonic mucosal biopsies from dogs fed CD or WD over the treatment schedule. (a) Period CD1, (b) period WD, and (c) period CD2. The expression of NF‐ĸB is observed primarily within the epithelial cells lining the colonic crypts. Note the up‐regulated expression in colonic tissues of dogs fed WD in comparison to tissues from dogs fed during periods CD1 and CD2. All images at 40× magnification. (B) Quantitative expression of NF‐κB in colonic mucosal biopsies from dogs fed CD or WD over the treatment schedule. Expressions are quantified using ImageJ. ***Significantly different at *p* < 0.0005. **Significantly different at *p* < 0.005. Data are presented as median ± SD. CD1 = Control Diet Period 1; CD2 = Control Diet Period 2; ns = no significant difference; WD = Western diet.

**FIGURE 4:**
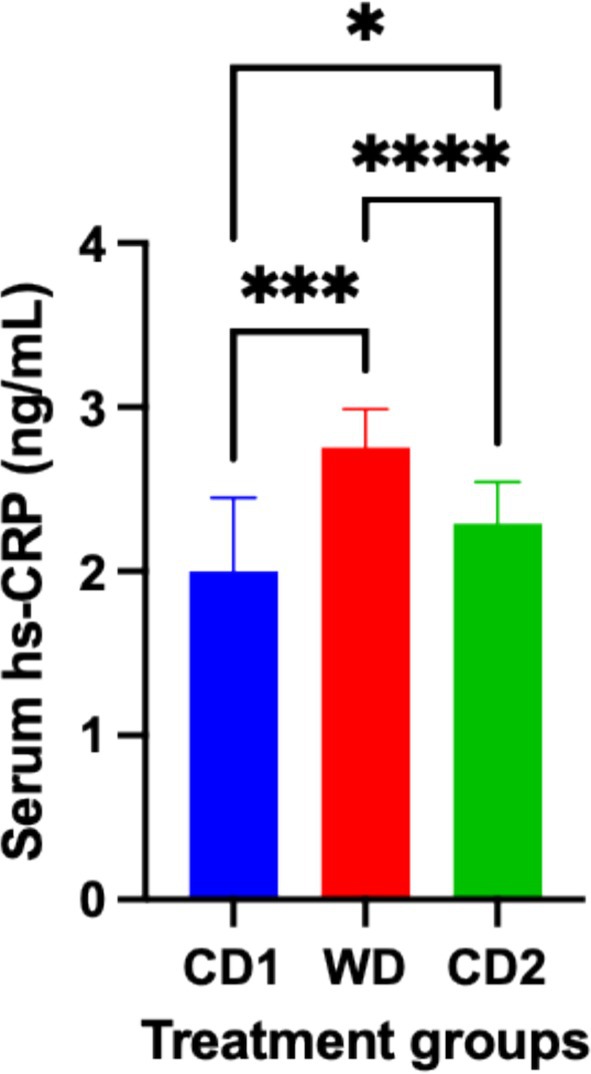
Serum high‐sensitivity C‐reactive protein (hs‐CRP) concentrations from dogs fed CD or WD over the treatment schedule. Serum concentrations are determined by ELISA. ****Significantly different at *p* < 0.0001. ***Significantly different at *p* = 0.0007. *Significantly different at *p* < 0.05. Data are presented as mean ± SD. CD1 = Control Diet Period 1; CD2 = Control Diet Period 2; ns = no significant difference; WD = Western diet.

**FIGURE 5:**
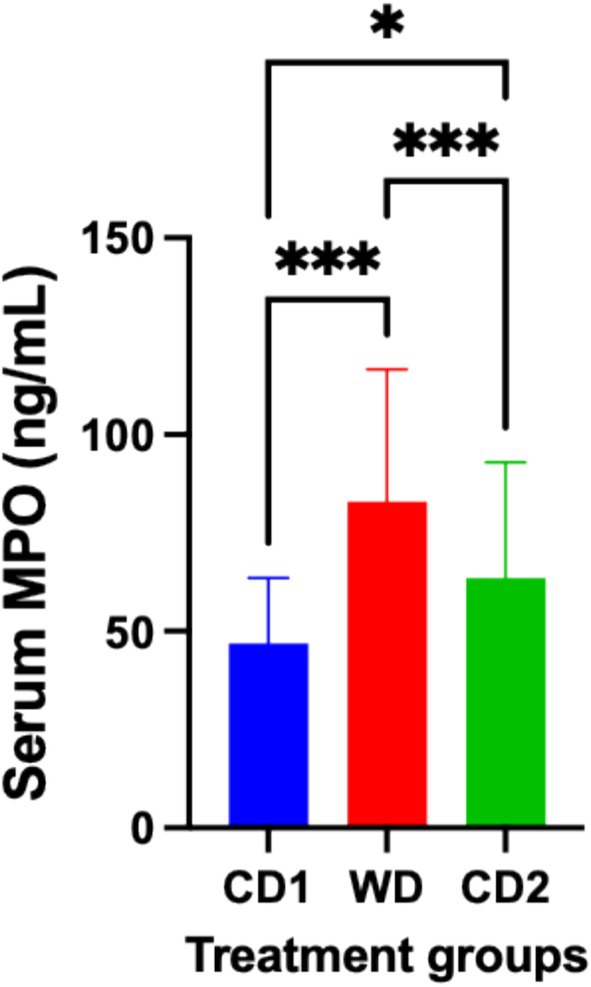
Serum myeloperoxidase (MPO) concentrations from dogs fed CD or WD over the treatment schedule. Serum concentrations are determined by ELISA. ***Significantly different at *p* < 0.0001. *Significantly different at *p* < 0.05. Data are presented as mean ± SD. CD1 = Control Diet Period 1; CD2 = Control Diet Period 2; ns = no significant difference; WD = Western diet.

**FIGURE 6:**
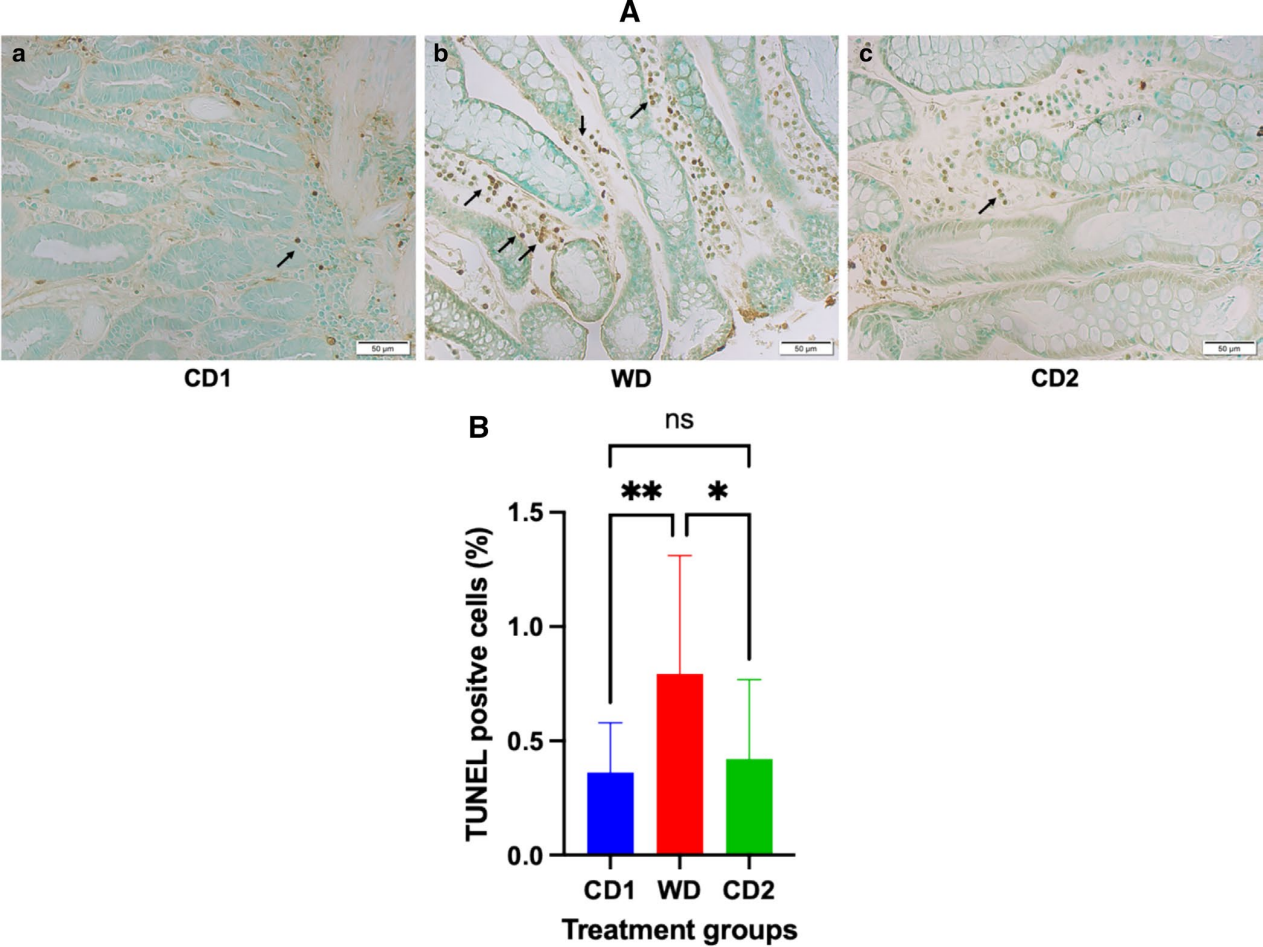
(A) TUNEL expression indicating apoptosis in colonic mucosal biopsies from dogs fed CD or WD over the treatment schedule. (a) Period CD1, (b) period WD, (c) period CD2. TUNEL‐positive mononuclear cells (lymphocytes) are found within the colonic lamina propria. Note the increased numbers of apoptotic lymphocytes observed in colonic tissues of dogs fed WD in comparison to tissues from dogs fed during periods CD1 and CD2. The arrows indicate cells that are TUNEL positive. (B) Quantitative analysis of TUNEL‐positive lymphocytes/mucosal area in colonic mucosal biopsies from dogs fed CD or WD over the treatment schedule. Numbers of apoptotic cells are quantified using ImageJ. Data are presented as mean ± SD. **Significantly different at *p* < 0.01. *Significantly different at *p* < 0.05. All images at ×40 magnification. CD1 = Control Diet Period 1; CD2 = Control Diet Period 2; TUNEL = terminal deoxynucleotidyl transferase dUTP nick end labeling; WD = Western diet.

Dynamic changes in inflammatory, oxidative, and apoptotic markers were observed in response to dietary transitions during the trial. The expression of NF‐ĸB in colonic tissues of dogs fed WD was significantly increased (*p* < 0.001) compared to colonic expression in dogs fed CD (NF‐ĸB area: CD1, 8.09% ± 3.3%; WD, 11.58% ± 3.4%; CD2 7.25% ± 3.8%; 95% CI; Figure 3A,B). Robust expression of NF‐ĸB was primarily localized to the colonic crypts, with less expression observed within the lamina propria of dogs fed either diet. At the completion of period C2, NF‐ĸB tissue expression decreased significantly (*p* = 0.0002) when compared to colonic expression in dogs fed WD. Dogs fed WD had a significantly increased (*p* = 0.0007) serum hs‐CRP concentration compared to the serum hs‐CRP concentration in dogs fed CD (CD1, 2.0 ± 0.4 ng/mL; WD, 2.76 ± 0.23 ng/mL; CD2, 2.29 ± 0.25 ng/mL; 95% CI). At the completion of period C2, the hs‐CRP serum concentration had decreased significantly (*p* < 0.0001) when compared to the concentration in WD but was not significantly different than the concentration in CD1 (Figure 4). Evidence of oxidative damage was present in dogs fed WD as the serum concentration of MPO was significantly increased (*p* = 0.0006) compared to the concentration observed in dogs fed either CD1 or CD2 (CD1, 46.98 ± 16.6 ng/mL; WD, 82.93 ± 33.6 ng/mL; CD2, 63.52 ± 29.5 ng/mL; 95% CI). Like the trend observed for hs‐CRP, the serum concentration of MPO was significantly decreased (*p* < 0.0001) when dogs transitioned from WD to CD2 but was different from the MPO serum concentration of dogs in CD1 (Figure 5). TUNEL‐positive cells, indicating lymphocyte apoptosis, were significantly increased in number (*p* = 0.009) in the lamina propria of dogs fed WD in comparison to their expression in the lamina propria of dogs fed CD (TUNEL‐positive cells: CD1, 0.36% ± 0.2%; WD, 0.79% ± 0.5%; CD2, 0.42% ± 0.3%; 95% CI). Dogs transitioning from WD to CD2 had significantly decreased (*p* = 0.01) numbers of TUNEL‐positive cells, like those observed in CD1 (Figure 6A,B).

**FIGURE 7:**
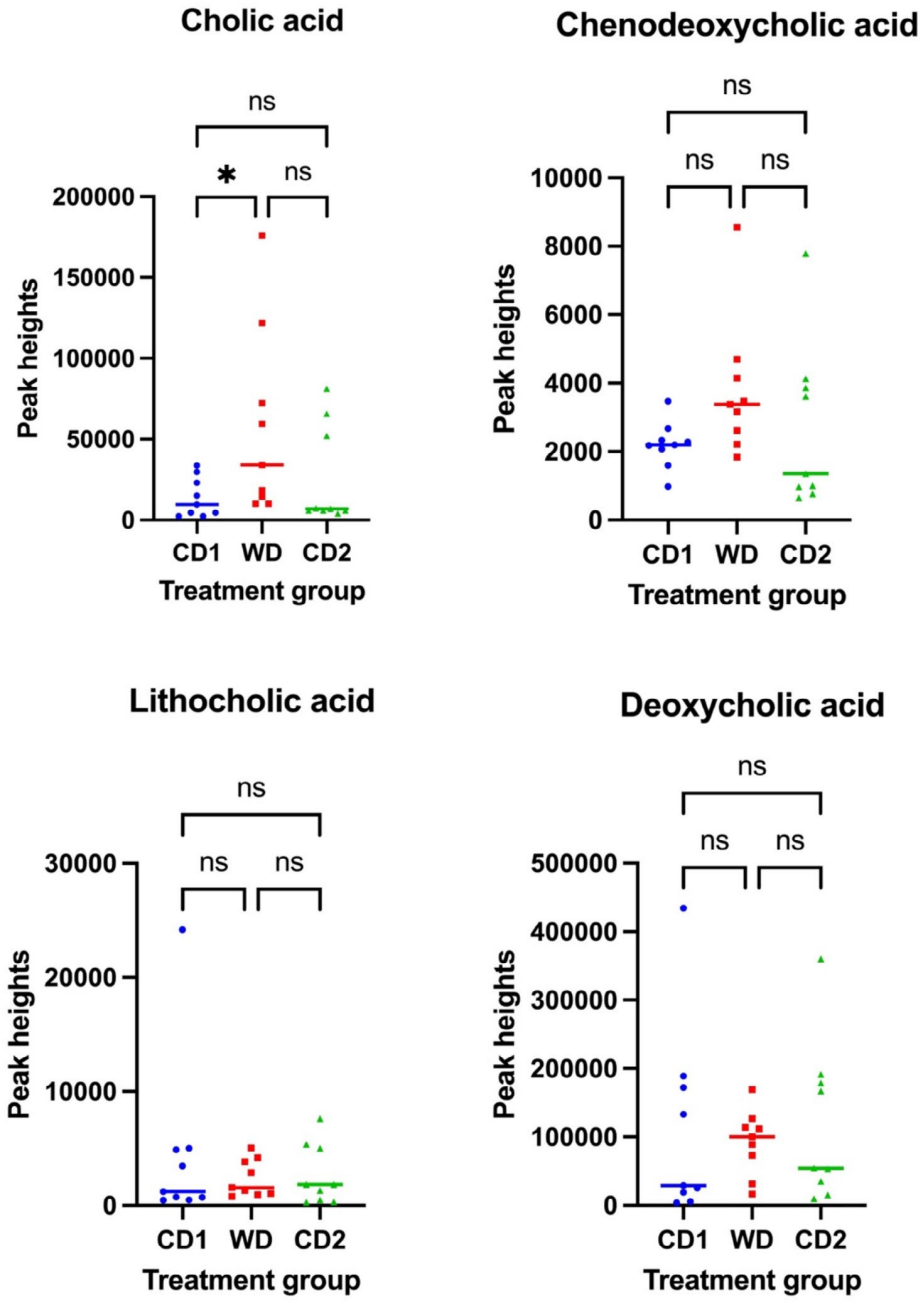
Relative concentrations of fecal unconjugated primary and secondary bile acids from dogs fed CD or WD over the treatment schedule. Data is grouped into primary (cholic acid and CDCAs) and secondary (lithocholic and DCAs) bile acids. The relative bile acid concentrations were determined using a dilutional gas chromatography‐mass spectrometry method. *Significantly different at *p* < 0.05. A non‐parametric statistical test, the Friedman test, was used for analyzing repeated measures data. Data are presented as median. CD1 = Control Diet Period 1; CD2 = Control Diet Period 2; ns = no significant difference; WD = Western diet.

Analysis of fUBA in dogs fed either diet showed a significant difference only in the excretion of one of the primary fUBA. Dogs fed WD showed increased relative concentrations of primary fUBA, with the CA increased significantly (median and interquartile range/IQR: CD1, 9505 (2384–33 788) peak heights; WD, 34 131 (10 113–175 909) peak heights; *p* < 0.05) in comparison to primary fUBA, CDCA. Following the diet transition from WD to CD2, the concentrations of both primary (CA and CDCA) and secondary fUBA (LCA and DCA) were not significantly altered once returned to the CD (Figure 7).

**FIGURE 8:**
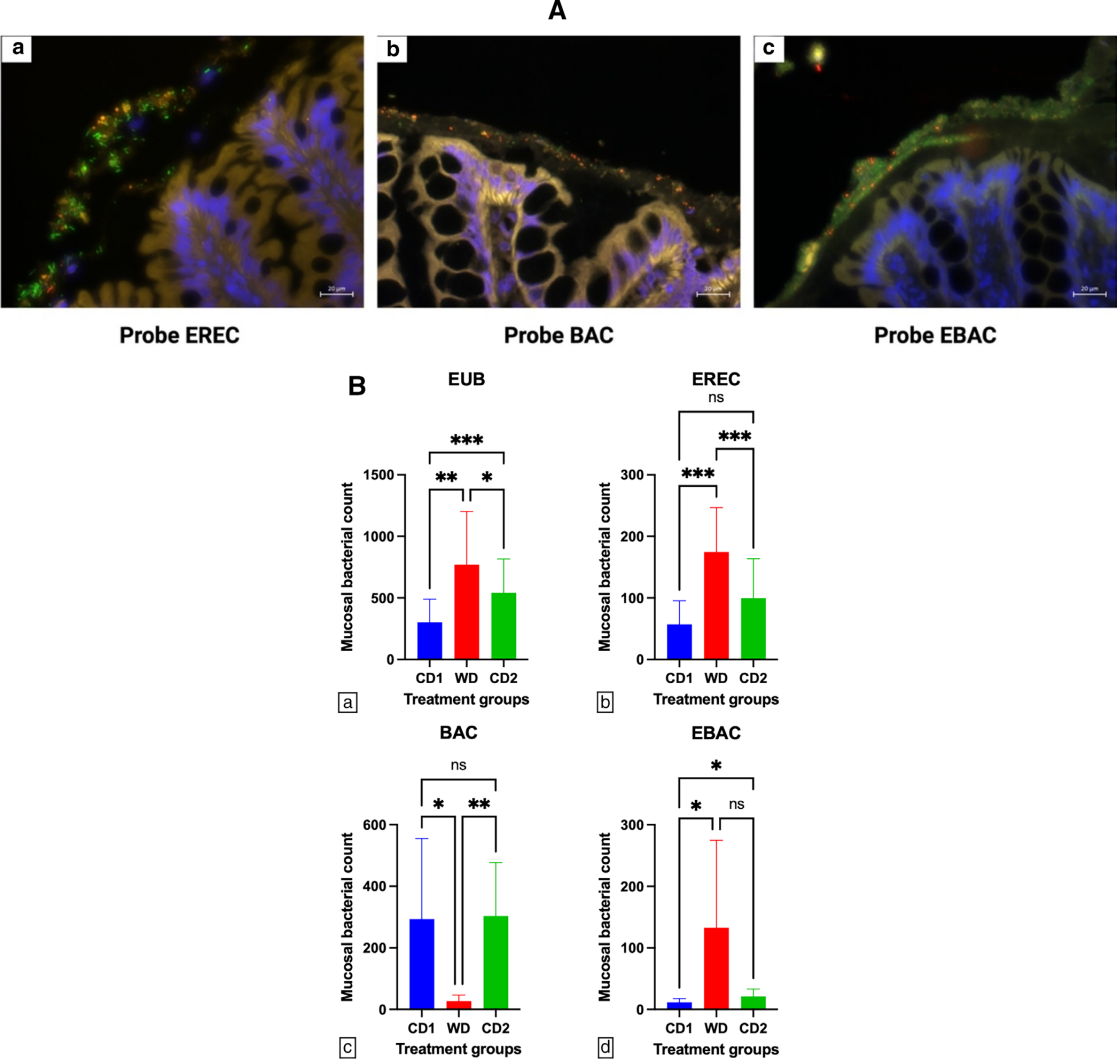
(A) Three‐color FISH identifies mucosal bacteria present in adherent mucus of colonic biopsies from dogs fed CD or WD over the treatment schedule. Specific bacterial groups Clostridia (EREC482), Bacteroides (BAC303), and Enterobacteriaceae (EBAC1790) hybridizing with Cy3 appear orange. All other bacteria hybridizing with the universal probe (EUB‐FITC) appear green. DAPI‐stained colonic mucosa with nuclei stain blue. (a) Period WD, EREC probe; (b) period WD, BAC probe; (c) period WD, EBAC probe. All images at ×40 magnification. (B) Numbers of mucosal bacteria in adherent mucus of colonic biopsies from dogs fed CD or WD over the treatment schedule. Data are expressed as mean ± standard deviation/mucosal area. (a) EUB = total bacteria, (b) EREC probe = Clostridia, (c) BAC probe = Bacteroides, and (d) EBAC probe = Enterobacteriaceae. ***Significantly different at *p* < 0.0005. **Significantly different at *p* < 0.005. *Significantly different at *p* < 0.05. Data are presented as mean ± SD. ns = no significant difference.

The colonic mucosal microbiota of dogs fed CD and WD was most abundant in adherent mucus. Few bacteria were observed in the free mucus or attached to the colonic epithelia. The total number of EUB‐positive bacteria was significantly increased (*p* < 0.001) in dogs fed WD when compared to dogs fed CD. Moreover, dogs fed WD had a significantly different spatial distribution of bacteria compared to dogs fed CD, with increased numbers of Clostridia (*p* = 0.0006) and Enterobacteriaceae (*p* < 0.05) but decreased numbers of Bacteroides (*p* = 0.03) found within adherent mucus (Figure 8A,B). The abundance ratio of mucosal Clostridia to Bacteroides of dogs fed WD compared to dogs fed CD1 and CD2 was 6.36–0.19, and 0.33, respectively.

**FIGURE 9:**
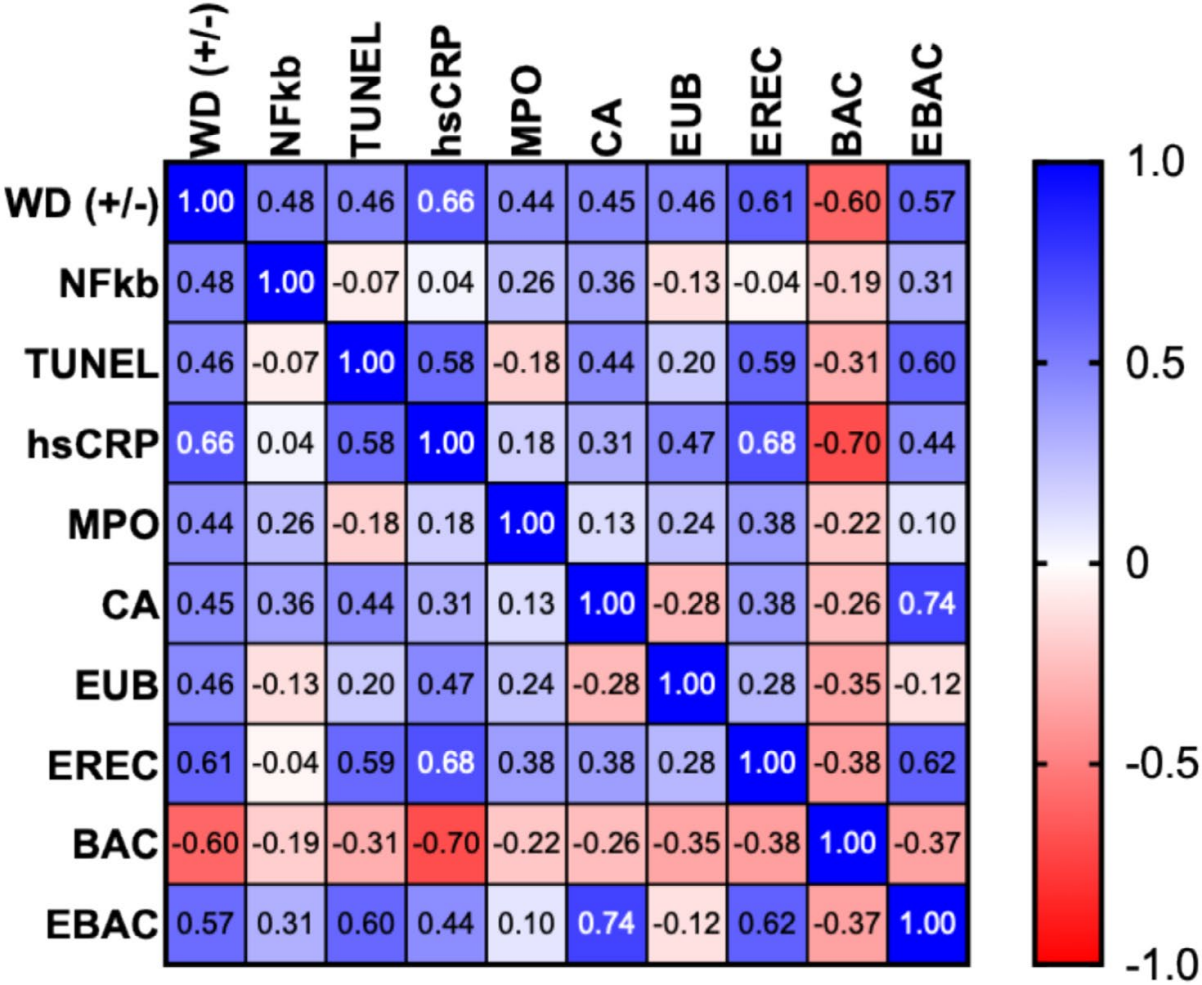
Heat map displaying Pearson's correlation coefficients between the effects of WD on inflammatory indices, oxidative damage, microbiome, and fecal bile acids (BA) in dogs. Minimal statistical significance was accepted at *p* < 0.05. Positive correlations are in blue, and negative correlations are in red. −, without; +, with; BAC, specific probes directed against Bacteroides, BAC303; CA, cholic acid; EBAC, specific probes directed against Enterobacteriaceae, EBAC1790; EREC, specific probes directed against *Clostridium*, EREC482; EUB, probe EUB338‐FITC for total bacterial counts; hs‐CRP, high‐sensitivity C‐reactive protein; MPO, myeloperoxidase; NF‐ĸB, nuclear factor ĸB; TUNEL, terminal deoxynucleotidyl transferase dUTP nick end labeling; WD, Western‐style diet.

In dogs fed WD, there was a positive correlation with inflammatory/apoptotic indices including NF‐ĸB (*p* = 0.007), serum hs‐CRP (*p* = 0.00008), numbers of lamina propria TUNEL‐positive cells (*p* = 0.01), and fecal CA concentration (*p* = 0.01). The correlation between TUNEL‐positive cells and serum hs‐CRP concentration was positive (*p* = 0.0008), while serum MPO activity showed a positive correlation with the numbers of mucosal Clostridia (*p* < 0.05; Figure 9).

## 4 Discussion

Studies in humans and animal models have shown that HFD and its influence on the intestinal microbiota are closely related to obesity [30, 31], metabolic disturbances [32, 33], and GI diseases [1, 34, 35]. While previous studies have demonstrated HFD‐induced effects on the canine fecal microbiome and metabolome, they have not demonstrated the causal association between WD, mucosal dysbiosis, BA dysmetabolism, and intestinal inflammation in clinically healthy dogs. In this study, we investigated how a diet transition from a CD to WD, and inversely, during a 16‐week dietary intervention trial influenced host inflammation, oxidative stress, apoptosis, mucosal microbiota, and fUBAs in clinically healthy dogs. Specifically, WD was associated with low‐grade mucosal (NF‐ĸB expression) and systemic (hs‐CRP) inflammation, increased serum MPO, mucosal lymphocyte apoptosis, mucosal dysbiosis, and fecal BA dysmetabolism compared with parameters assessed in canine biological samples from periods CD1 and CD2.

Other studies have examined how different dietary constituents influence the fecal microbiome in dogs. Healthy dogs fed a non‐digestible carbohydrate (fiber) enriched diet exhibit an intestinal microbiome characterized by three dominant bacterial phyla, Bacteroidetes, Firmicutes, and Fusobacteria [36, 37, 38]. Conversely, dogs fed an animal‐derived (primarily beef) diet showed different microbial shifts in feces, including decreased numbers of Lachnospiraceae and increased numbers of *C.perfringens* [39, 40, 41]. Other studies have shown that HFD caused no significant shifts in the fecal microbiota regarding alpha and beta diversity [13] or demonstrated a significant shift in fecal microbiota diversity [14, 42, 43] Similarly, rodent studies have shown that HFDs are generally associated with decreased overall microbial abundance and diversity, with a shift from Bacteroidetes to Firmicutes [32, 44, 45]. Importantly, these changes in microbial composition and spatial redistribution along the gut mucous layer can lead to impaired intestinal barrier integrity (due to reduced tight junction protein expression), intestinal inflammation, and increased disease risk, including obesity [1, 32, 43].

Knowledge of the mucosal microbiota has lagged behind that of the fecal microbiota due to differences in the ease of collection of biological samples. This study reports the effects of WD on the canine colonic mucosal microbiota. Using FISH probes targeting key bacterial groups within endoscopic biopsies, we showed that dogs transitioning from a CD to WD, and vice versa, showed significant differences in the number of total mucosal bacteria and shifts in microbiota composition. Specifically, the mucosal abundance of total bacteria, Clostridia, and Enterobacteriaceae increased, while the abundance of Bacteroides decreased in dogs fed WD as compared to CD. A common dysbiosis metric used in obesity studies of humans, mice, and dogs is the detection of significant differences in the abundance of Firmicutes and Bacteroides [32, 44, 45]. Coelho et al. [46] previously reported an increase in the fecal Firmicutes: Bacteroides ratio of dogs fed a high‐protein, low‐carbohydrate ration. Application of this metric to the adherent mucosal microbiota of dogs in the present study showed that dogs fed WD had an increased Clostridia: Bacteroides ratio of 6.36 compared to ratios of 0.19 and 0.33 in dogs fed during transition periods CD1 and CD2, respectively. The significant shifts in the Clostridia: Bacteroides abundance observed with dietary transition to WD confirm the plasticity of the intestinal microbiome in response to changes in macronutrient composition [39, 47]. The Clostridia: Bacteroides ratio was utilized as Clostridia were the only members of the Firmicutes phylum measured.

Intestinal inflammation is a consequence of WD in humans and rodent models. WD promotes dysbiosis with increased abundance of pro‐inflammatory microbes, epithelial barrier dysfunction, and increased intestinal permeability with leakage of bacterial metabolites into the bloodstream that produce low‐grade systemic inflammation [48, 49, 50, 51]. HFD/WD consumption increases the abundance of Proteobacteria [52] which are gram‐negative LPS‐containing bacteria in the order Enterobacteriaceae [53]. HFD/WD‐driven dysbiosis with increased LPS production can activate the TLR4/NF‐κB pathway to stimulate the production of proinflammatory cytokines TNF‐α, IL‐1β, and IL‐6, causing intestinal inflammation [54, 55]. Other investigations have shown that saturated fatty acids, themselves, can exert a similar molecular effect to LPS and activate TLR4 to stimulate proinflammatory cytokine secretion, perturb mucosal homeostasis, and disrupt cellular metabolism [56, 57]. The present study suggests the ability of WD to induce local and systemic inflammation in clinically healthy dogs. The expression of NF‐κB in colonic tissues of dogs fed WD was increased compared with dogs fed CD. NF‐κB expression was most evident in mucosal regions around colonic crypts but was also found within cells in the lamina propria. Moreover, the elevated serum hs‐CRP concentration in dogs fed WD was a sensitive indicator of systemic inflammation in response to mucosal proinflammatory stimulation [58]. The blood concentration of CRP can change rapidly, within 4–6 h, following an inflammatory stimulus, with maximum concentration after 1–2 days, which broadly reflects the severity of tissue inflammation [59]. Increased tissue expression of NF‐κB [60] and elevated serum concentration of CRP [61, 62, 63] have been associated with intestinal inflammation in dogs with IBD (now re‐classified as immunosuppressant‐responsive enteropathy, which is a phenotype of chronic inflammatory enteropathy [CIE]) [64].

Oxidative stress is another mechanism where WD serves as a potential proinflammatory stimulus promoting low‐grade intestinal inflammation. In support of this notion and using a rodent model, Gulhane et al. [65] observed that HFD induced increased expression of numerous genes considered as markers of endoplasmic reticulum (ER)/oxidative stress. Moreover, increased fat consumption in mice may trigger mitochondrial β‐oxidation of free fatty acids that increase the concentration of reactive oxygen species in tissues, which are pro‐inflammatory [66]. Finally, HFD/WD may result in the loss of XIAP (X‐linked inhibitor of apoptosis) function, which increases inflammasome activity, oxidative stress, and apoptosis [67]. In the current study, increased concentration of serum MPO was observed in dogs fed WD as compared to dogs fed CD. WD consumption was also associated with a marked increase in the number of apoptotic cells (likely CD3+ T lymphocytes [68]) found within the colonic lamina propria. Increased apoptosis of lamina propria lymphocytes in dogs fed WD may represent a compensatory homeostatic mechanism to downregulate mucosal inflammation [69]. It is most likely that the cause of increased lamina propria apoptotic activity in WD‐fed dogs is multifactorial, where oxidative stress, inflammation with activation of the NF‐ĸB pathway, and disturbances in BA metabolism (see below) contribute synergistically.

BAs are required for the digestion and absorption of lipids and fat‐soluble vitamins, but they also serve important roles in maintaining mucosal homeostasis [1, 70, 71]. Following their synthesis from cholesterol in the liver, primary BA (CA and CDCA) are conjugated to glycine or taurine and modified by the intestinal microbiota into secondary BA (LCA and DCA) [72, 73]. Most (95%) of the conjugated BA are then absorbed in the distal ileum by the apical sodium‐dependent BA transporter (ASBT) [74] before returning to the liver for re‐secretion, resulting in enterohepatic circulation [75]. BA undergo several transformations by bacteria in the colon, including deconjugation and the formation of secondary BA. Therefore, a dynamic balance between BA homeostasis and the intestinal microbiota exists that, when disturbed, can result in dysbiosis and an altered BA profile [70, 76, 77]. Several GI inflammatory pathologies are associated with both microbial imbalances and BA dysmetabolism, including IBD in people [78] and CIE in dogs [22, 79, 80].

Diet can have considerable influence on microbial composition and the production of bacterial metabolites, including BA. Mice fed HFD have an impaired intestinal mucosal barrier and a modified BA profile characterized by increased concentrations of primary (DCA) BA and decreased concentrations of the cytoprotective tertiary BA, UDCA [81]. Another study found that HFD induced the expansion of the pathobiont *Bilophilia wadsworthia*, a sulfur‐producing and bile‐tolerant microbe that provokes a robust Th1 immune response causing colitis in IL‐10 deficient mice [82].

Few studies have reported disturbances in fecal BA composition in dogs fed HFD. One study involving overweight adult female dogs fed a high‐protein, high‐fiber weight loss diet found that fecal DCA concentrations decreased, fecal secondary BA concentrations tended to decrease, and fecal UDCA concentrations increased with restricted feeding and weight loss [83]. In another study, adult client‐owned dogs fed a BARF diet failed to show alterations in fecal BA compared to dogs fed a commercial diet [14]. Finally, changes in fecal BA concentrations were investigated in healthy client‐owned dogs before, during, and after HFD. Results showed that relative concentrations of the fecal secondary BA, DCA, were increased in dogs fed HFD but not in these same dogs fed a commercial diet. While the median concentrations of primary BAs, CA, and CDCA, were increased in dogs fed HFD, only the taurine‐conjugated BAs were significantly elevated compared with commercial diet samples [15]. Results in dogs from the present study show similarities and differences to these earlier reports. In dogs fed WD, only primary BA was significantly increased with relative fecal concentrations of the primary BA, CA, reaching significance compared to samples obtained in C1 and C2. The concentration of the secondary BA, DCA, was greater in dogs fed WD than the concentration of LCA but not significantly. Diet transition from WD to CD2 produced insignificant alterations of BA concentration for four primary and secondary BAs, including CA, CDCA, LCA, and DCA. We investigated the four BAs in the present study because they are the most abundant, most well studied, and most important regarding mucosal homeostasis in dogs [22, 79, 80, 84].

A limitation of this study is that the diets formulated for dogs contained all essential nutrients at the required amount for canine adult maintenance per NRC, which is not typically the case for WD in people where they lack recommended vitamins and minerals. In addition, although the diet fed in this study mimicked the more widely understood WD, this was only in macronutrient composition and not in the many other aspects, such as high salt or fructose sugar content, which may be of importance for the long‐term effects of this diet. In addition, dogs in this study were housed as a closed colony in a clean environment as compared to pet dogs raised in environmental conditions that cohabitated with humans. Finally, the use of the Clostridia: Bacteroidetes ratio was required as the entire Firmicutes phylum was not measured in the present study.

To conclude, we present evidence that WD fed short term to clinically healthy dogs promotes colonic dysbiosis and increases in mucosal NF‐ĸB expression, serum hs‐CRP, serum MPO, and colonic lamina propria lymphocyte apoptosis. Fecal primary and secondary BA concentrations were modified in response to dietary transition from the CD to WD, and vice versa, with increased concentrations of CA observed in dogs fed WD. The dog represents a good preclinical model for intestinal health to humans due to its similarities in GI anatomy and physiology, dietary patterns, metabolic processes, and pathology of intestinal diseases [36].

## Disclosure

Authors declare no off‐label use of antimicrobials.

## Ethics Statement

Institutional Animal Care and Use Committee (IACUC) approval IACUC‐19‐337. All experiments were performed in accordance with relevant guidelines and regulations of IACUC as required by US federal regulations. The study is reported in accordance with the ARRIVE guidelines (https://arriveguidelines.org, accessed on August 3, 2020). Authors declare human ethics approval was not needed.

## Conflicts of Interest

The authors declare no conflicts of interest.

## References

[1] I. J. Malesza , M. Malesza , J. Walkowiak , et al., “High‐Fat, Western‐Style Diet, Systemic Inflammation, and Gut Microbiota: A Narrative Review,” Cells 10, no. 11 (2021): 3164, 10.3390/cells10113164. 34831387 PMC8619527

[2] L. Cordain , S. B. Eaton , A. Sebastian , et al., “Origins and Evolution of the Western Diet: Health Implications for the 21st Century,” American Journal of Clinical Nutrition 81 (2005): 341–354. 15699220 10.1093/ajcn.81.2.341

[3] A. Christ , M. Lauterbach , and E. Latz , “Western Diet and the Immune System: An Inflammatory Connection,” Immunity 51 (2019): 794–811. 31747581 10.1016/j.immuni.2019.09.020

[4] D. Statovci , M. Aguilera , J. MacSharry , and S. Melgar , “The Impact of Western Diet and Nutrients on the Microbiota and Immune Response at Mucosal Interfaces,” Frontiers in Immunology 8 (2017). 10.3389/fimmu.2017.00838 PMC5532387 28804483

[5] O. Varlamov , “Western‐style diet, sex steroids and metabolism,” Biochimica et Biophysica Acta ‐ Molecular Basis of Disease 1863 (2017): 1147–1155. 27264336 10.1016/j.bbadis.2016.05.025

[6] D. Mozaffarian , T. Hao , E. B. Rimm , et al., “Changes in Diet and Lifestyle and Long‐Term Weight Gain in Women and Men,” New England Journal of Medicine 364 (2011): 2392–2404. 21696306 10.1056/NEJMoa1014296 PMC3151731

[7] C. S. C. Yip , W. Lam , and R. Fielding , “A Summary of Meat Intakes and Health Burdens,” European Journal of Clinical Nutrition 72 (2018): 18–29. 28792013 10.1038/ejcn.2017.117

[8] A. Pan , Q. Sun , A. M. Bernstein , et al., “Changes in Red Meat Consumption and Subsequent Risk of Type 2 Diabetes Mellitus: Three Cohorts of US Men and Women,” JAMA Internal Medicine 173 (2013): 1328–1335. 23779232 10.1001/jamainternmed.2013.6633 PMC3847817

[9] A. Pan , Q. Sun , A. M. Bernstein , et al., “Red Meat Consumption and Mortality: Results From 2 Prospective Cohort Studies,” Archives of Internal Medicine 172 (2012): 555–563. 22412075 10.1001/archinternmed.2011.2287 PMC3712342

[10] D. S. Chan , R. Lau , D. Aune , et al., “Red and Processed Meat and Colorectal Cancer Incidence: Meta‐Analysis of Prospective Studies,” PLoS One 6 (2011): e20456. 21674008 10.1371/journal.pone.0020456 PMC3108955

[11] A. Manzel , D. N. Muller , D. A. Hafler , et al., “Role of “Western Diet” in Inflammatory Autoimmune Diseases,” Current Allergy and Asthma Reports 14 (2014): 404. 24338487 10.1007/s11882-013-0404-6 PMC4034518

[12] L. C. Graham , J. M. Harder , I. Soto , W. N. de Vries , S. W. M. John , and G. R. Howell , “Chronic Consumption of a Western Diet Induces Robust Glial Activation in Aging Mice and in a Mouse Model of Alzheimer's Disease,” Scientific Reports 6 (2016): 21568. 26888450 10.1038/srep21568 PMC4757836

[13] L. R. Kilburn , L. R. Koester , S. Schmitz‐Esser , N. V. L. Serão , and M. C. Rossoni Serão , “High‐Fat Diets Led to OTU‐Level Shifts in Fecal Samples of Healthy Adult Dogs,” Frontiers in Microbiology 11 (2020): 564160. 33363518 10.3389/fmicb.2020.564160 PMC7752866

[14] M. Schmidt , S. Unterer , J. S. Suchodolski , et al., “The Fecal Microbiome and Metabolome Differs Between Dogs Fed Bones and Raw Food (BARF) Diets and Dogs Fed Commercial Diets,” PLoS One 13 (2018): e0201279. 30110340 10.1371/journal.pone.0201279 PMC6093636

[15] K. M. V. Herstad , H. T. Rønning , A. M. Bakke , L. Moe , and E. Skancke , “Changes in the Faecal Bile Acid Profile in Dogs Fed Dry Food vs High Content of Beef: A Pilot Study,” Acta Veterinaria Scandinavica 60 (2018): 29. 29751815 10.1186/s13028-018-0383-7 PMC5948804

[16] J. Y. Li , M. Gillilland, 3rd , A. A. Lee , et al., “Secondary Bile Acids Mediate High‐Fat Diet‐Induced Upregulation of R‐Spondin 3 and Intestinal Epithelial Proliferation,” JCI Insight 7, no. 19 (2022): e148309, 10.1172/jci.insight.148309. 36099053 PMC9675439

[17] B. J. Byiers , J. Reichle , and F. J. Symons , “Single‐Subject Experimental Design for Evidence‐Based Practice,” American Journal of Speech‐Language Pathology 21 (2012): 397–414. 23071200 10.1044/1058-0360(2012/11-0036) PMC3992321

[18] A. E. Jergens , M. D. Willard , and K. Allenspach , “Maximizing the Diagnostic Utility of Endoscopic Biopsy in Dogs and Cats With Gastrointestinal Disease,” Veterinary Journal 214 (2016): 50–60. 27387727 10.1016/j.tvjl.2016.04.008

[19] A. E. Jergens , R. B. Evans , M. Ackermann , et al., “Design of a Simplified Histopathologic Model for Gastrointestinal Inflammation in Dogs,” Veterinary Pathology 51 (2014): 946–950. 24280943 10.1177/0300985813511123

[20] A. R. Crowe and W. Yue , “Semi‐Quantitative Determination of Protein Expression Using Immunohistochemistry Staining and Analysis: An Integrated Protocol,” Bio‐Protocol 9, no. 24 (2019): e3465, 10.21769/BioProtoc.3465. 31867411 PMC6924920

[21] D. K. Sahoo , K. Allenspach , J. P. Mochel , et al., “Synbiotic‐IgY Therapy Modulates the Mucosal Microbiome and Inflammatory Indices in Dogs With Chronic Inflammatory Enteropathy: A Randomized, Double‐Blind, Placebo‐Controlled Study,” Veterinary Sciences 10, no. 1 (2022): 25. 36669027 10.3390/vetsci10010025 PMC9867299

[22] B. C. Guard , J. B. Honneffer , A. E. Jergens , et al., “Longitudinal Assessment of Microbial Dysbiosis, Fecal Unconjugated Bile Acid Concentrations, and Disease Activity in Dogs With Steroid‐Responsive Chronic Inflammatory Enteropathy,” Journal of Veterinary Internal Medicine 33 (2019): 1295–1305. 30957301 10.1111/jvim.15493 PMC6524081

[23] A. E. Jergens , M. Pressel , J. Crandell , et al., “Fluorescence In Situ Hybridization Confirms Clearance of Visible *Helicobacter* Spp. Associated With Gastritis in Dogs and Cats,” Journal of Veterinary Internal Medicine 23 (2009): 16–23. 19175715 10.1111/j.1939-1676.2008.0211.x

[24] R. White , T. Atherly , B. Guard , et al., “Randomized, Controlled Trial Evaluating the Effect of Multi‐Strain Probiotic on the Mucosal Microbiota in Canine Idiopathic Inflammatory Bowel Disease,” Gut Microbes 8 (2017): 451–466. 28678609 10.1080/19490976.2017.1334754 PMC5628651

[25] A. H. Franks , H. J. Harmsen , G. C. Raangs , et al., “Variations of Bacterial Populations in Human Feces Measured by Fluorescent In Situ Hybridization With Group‐Specific 16S rRNA‐Targeted Oligonucleotide Probes,” Applied and Environmental Microbiology 64 (1998): 3336–3345. 9726880 10.1128/aem.64.9.3336-3345.1998 PMC106730

[26] W. Manz , R. Amann , W. Ludwig , et al., “Application of a Suite of 16S rRNA‐Specific Oligonucleotide Probes Designed to Investigate Bacteria of the Phylum Cytophaga‐Flavobacter‐Bacteroides in the Natural Environment,” Microbiology 142 (1996): 1097–1106. 8704951 10.1099/13500872-142-5-1097

[27] L. K. Poulsen , F. Lan , C. S. Kristensen , P. Hobolth , S. Molin , and K. A. Krogfelt , “Spatial Distribution of *Escherichia coli* in the Mouse Large Intestine Inferred From rRNA In Situ Hybridization,” Infection and Immunity 62 (1994): 5191–5194. 7927805 10.1128/iai.62.11.5191-5194.1994 PMC303247

[28] J. B. Honneffer , Y. Minamoto , and J. S. Suchodolski , “Microbiota Alterations in Acute and Chronic Gastrointestinal Inflammation of Cats and Dogs,” World Journal of Gastroenterology 20 (2014): 16489–16497. 25469017 10.3748/wjg.v20.i44.16489 PMC4248192

[29] J. S. Suchodolski , S. E. Dowd , V. Wilke , J. M. Steiner , and A. E. Jergens , “16S rRNA Gene Pyrosequencing Reveals Bacterial Dysbiosis in the Duodenum of Dogs With Idiopathic Inflammatory Bowel Disease,” PLoS One 7 (2012): e39333. 22720094 10.1371/journal.pone.0039333 PMC3376104

[30] B. W. Parks , E. Nam , E. Org , et al., “Genetic Control of Obesity and Gut Microbiota Composition in Response to High‐Fat, High‐Sucrose Diet in Mice,” Cell Metabolism 17 (2013): 141–152. 23312289 10.1016/j.cmet.2012.12.007 PMC3545283

[31] P. J. Turnbaugh , V. K. Ridaura , J. J. Faith , et al., “The Effect of Diet on the Human Gut Microbiome: A Metagenomic Analysis in Humanized Gnotobiotic Mice,” Science Translational Medicine 1 (2009): 6ra14. 10.1126/scitranslmed.3000322 PMC2894525 20368178

[32] E. A. Murphy , K. T. Velazquez , and K. M. Herbert , “Influence of High‐Fat Diet on Gut Microbiota: A Driving Force for Chronic Disease Risk,” Current Opinion in Clinical Nutrition and Metabolic Care 18 (2015): 515–520. 26154278 10.1097/MCO.0000000000000209 PMC4578152

[33] A. Tvarijonaviciute , J. J. Ceron , S. L. Holden , et al., “Obesity‐Related Metabolic Dysfunction in Dogs: A Comparison With Human Metabolic Syndrome,” BMC Veterinary Research 8 (2012): 147. 22929809 10.1186/1746-6148-8-147 PMC3514388

[34] M. Zhang and X. J. Yang , “Effects of a High Fat Diet on Intestinal Microbiota and Gastrointestinal Diseases,” World Journal of Gastroenterology 22 (2016): 8905–8909. 27833381 10.3748/wjg.v22.i40.8905 PMC5083795

[35] Y. Tong , H. Gao , Q. Qi , et al., “High Fat Diet, Gut Microbiome and Gastrointestinal Cancer,” Theranostics 11 (2021): 5889–5910. 33897888 10.7150/thno.56157 PMC8058730

[36] K. S. Swanson , S. E. Dowd , J. S. Suchodolski , et al., “Phylogenetic and Gene‐Centric Metagenomics of the Canine Intestinal Microbiome Reveals Similarities With Humans and Mice,” ISME Journal 5 (2011): 639–649. 20962874 10.1038/ismej.2010.162 PMC3105739

[37] T. Vanhoutte , G. Huys , E. De Brandt , et al., “Molecular Monitoring and Characterization of the Faecal Microbiota of Healthy Dogs During Fructan Supplementation,” FEMS Microbiology Letters 249 (2005): 65–71. 15979820 10.1016/j.femsle.2005.06.003

[38] I. S. Middelbos , B. M. Vester Boler , A. Qu , B. A. White , K. S. Swanson , and G. C. Fahey , “Phylogenetic Characterization of Fecal Microbial Communities of Dogs Fed Diets With or Without Supplemental Dietary Fiber Using 454 Pyrosequencing,” PLoS One 5 (2010): e9768. 20339542 10.1371/journal.pone.0009768 PMC2842427

[39] I. Hang , T. Rinttila , J. Zentek , et al., “Effect of High Contents of Dietary Animal‐Derived Protein or Carbohydrates on Canine Faecal Microbiota,” BMC Veterinary Research 8 (2012): 90. 22735212 10.1186/1746-6148-8-90 PMC3464166

[40] J. Zentek , “Influence of Diet Composition on the Microbial Activity in the Gastro‐Intestinal Tract of Dogs. II. Effects on the Microflora in the Ileum Chyme,” Journal of Animal Physiology and Animal Nutrition 74 (1995): 53–61.

[41] J. Zentek , S. Fricke , M. Hewicker‐Trautwein , B. Ehinger , G. Amtsberg , and C. Baums , “Dietary Protein Source and Manufacturing Processes Affect Macronutrient Digestibility, Fecal Consistency, and Presence of Fecal *Clostridium perfringens* in Adult Dogs,” Journal of Nutrition 134 (2004): 2158s–2161s. 15284426 10.1093/jn/134.8.2158S

[42] K. M. V. Herstad , K. Gajardo , A. M. Bakke , et al., “A Diet Change From Dry Food to Beef Induces Reversible Changes on the Faecal Microbiota in Healthy, Adult Client‐Owned Dogs,” BMC Veterinary Research 13 (2017): 147. 28558792 10.1186/s12917-017-1073-9 PMC5450340

[43] A. Moinard , C. Payen , K. Ouguerram , et al., “Effects of High‐Fat Diet at Two Energetic Levels on Fecal Microbiota, Colonic Barrier, and Metabolic Parameters in Dogs,” Frontiers in Veterinary Science 7 (2020): 566282. 33102570 10.3389/fvets.2020.566282 PMC7545960

[44] M. A. Hildebrandt , C. Hoffmann , S. A. Sherrill‐Mix , et al., “High‐Fat Diet Determines the Composition of the Murine Gut Microbiome Independently of Obesity,” Gastroenterology 137 (2009): 1716‐24.e1‐2. 19706296 10.1053/j.gastro.2009.08.042 PMC2770164

[45] C. Zhang , M. Zhang , X. Pang , Y. Zhao , L. Wang , and L. Zhao , “Structural Resilience of the Gut Microbiota in Adult Mice Under High‐Fat Dietary Perturbations,” ISME Journal 6 (2012): 1848–1857. 22495068 10.1038/ismej.2012.27 PMC3446802

[46] L. P. Coelho , J. R. Kultima , P. I. Costea , et al., “Similarity of the Dog and Human Gut Microbiomes in Gene Content and Response to Diet,” Microbiome 6 (2018): 72. 29669589 10.1186/s40168-018-0450-3 PMC5907387

[47] J. M. Simpson , B. Martineau , W. E. Jones , J. M. Ballam , and R. I. Mackie , “Characterization of Fecal Bacterial Populations in Canines: Effects of Age, Breed and Dietary Fiber,” Microbial Ecology 44 (2002): 186–197. 12087428 10.1007/s00248-002-0001-z

[48] A. Agus , J. Denizot , J. Thévenot , et al., “Western Diet Induces a Shift in Microbiota Composition Enhancing Susceptibility to Adherent‐Invasive *E. coli* Infection and Intestinal Inflammation,” Scientific Reports 6 (2016): 19032. 26742586 10.1038/srep19032 PMC4705701

[49] M. Crawford , C. Whisner , L. Al‐Nakkash , et al., “Six‐Week High‐Fat Diet Alters the Gut Microbiome and Promotes Cecal Inflammation, Endotoxin Production, and Simple Steatosis Without Obesity in Male Rats,” Lipids 54 (2019): 119–131. 30860608 10.1002/lipd.12131

[50] S. J. Kim , S. E. Kim , A. R. Kim , S. Kang , M. Y. Park , and M. K. Sung , “Dietary Fat Intake and Age Modulate the Composition of the Gut Microbiota and Colonic Inflammation in C57BL/6J Mice,” BMC Microbiology 19 (2019): 193. 31429703 10.1186/s12866-019-1557-9 PMC6701133

[51] S. Ding , M. M. Chi , B. P. Scull , et al., “High‐Fat Diet: Bacteria Interactions Promote Intestinal Inflammation Which Precedes and Correlates With Obesity and Insulin Resistance in Mouse,” PLoS One 5 (2010): e12191. 20808947 10.1371/journal.pone.0012191 PMC2922379

[52] N. R. Shin , T. W. Whon , and J. W. Bae , “Proteobacteria: Microbial Signature of Dysbiosis in Gut Microbiota,” Trends in Biotechnology 33 (2015): 496–503. 26210164 10.1016/j.tibtech.2015.06.011

[53] J. R. Mujico , G. C. Baccan , A. Gheorghe , L. E. Díaz , and A. Marcos , “Changes in Gut Microbiota due to Supplemented Fatty Acids in Diet‐Induced Obese Mice,” British Journal of Nutrition 110 (2013): 711–720. 23302605 10.1017/S0007114512005612

[54] A. Schaeffler , P. Gross , R. Buettner , et al., “Fatty Acid‐Induced Induction of Toll‐Like Receptor‐4/Nuclear Factor‐kappaB Pathway in Adipocytes Links Nutritional Signalling With Innate Immunity,” Immunology 126 (2009): 233–245. 18624726 10.1111/j.1365-2567.2008.02892.x PMC2632685

[55] S. W. Wong , M. J. Kwon , A. M. Choi , et al., “Fatty Acids Modulate Toll‐Like Receptor 4 Activation Through Regulation of Receptor Dimerization and Recruitment Into Lipid Rafts in a Reactive Oxygen Species‐Dependent Manner,” Journal of Biological Chemistry 284 (2009): 27384–27392. 19648648 10.1074/jbc.M109.044065 PMC2785667

[56] K. Ohashi , R. Shibata , T. Murohara , and N. Ouchi , “Role of Anti‐Inflammatory Adipokines in Obesity‐Related Diseases,” Trends in Endocrinology and Metabolism 25 (2014): 348–355. 24746980 10.1016/j.tem.2014.03.009

[57] H. Shi , M. V. Kokoeva , K. Inouye , et al., “TLR4 Links Innate Immunity and Fatty Acid‐Induced Insulin Resistance,” Journal of Clinical Investigation 116 (2006): 3015–3025. 17053832 10.1172/JCI28898 PMC1616196

[58] R. M. Heilmann and J. M. Steiner , “Clinical Utility of Currently Available Biomarkers in Inflammatory Enteropathies of Dogs,” Journal of Veterinary Internal Medicine 32 (2018): 1495–1508. 30222209 10.1111/jvim.15247 PMC6189362

[59] K. Malin and O. Witkowska‐Piłaszewicz , “C‐Reactive Protein as a Diagnostic Marker in Dogs: A Review,” Animals 12, no. 20 (2022): 2888, 10.3390/ani12202888. 36290272 PMC9598812

[60] N. Luckschander , J. A. Hall , F. Gaschen , et al., “Activation of Nuclear Factor‐KappaB in Dogs With Chronic Enteropathies,” Veterinary Immunology and Immunopathology 133 (2010): 228–236. 19740552 10.1016/j.vetimm.2009.08.014

[61] A. E. Jergens , C. A. Schreiner , D. E. Frank , et al., “A Scoring Index for Disease Activity in Canine Inflammatory Bowel Disease,” Journal of Veterinary Internal Medicine 17 (2003): 291–297. 12774968 10.1111/j.1939-1676.2003.tb02450.x

[62] C. C. Otoni , R. M. Heilmann , M. García‐Sancho , et al., “Serologic and Fecal Markers to Predict Response to Induction Therapy in Dogs With Idiopathic Inflammatory Bowel Disease,” Journal of Veterinary Internal Medicine 32 (2018): 999–1008. 29624721 10.1111/jvim.15123 PMC5980281

[63] J. H. Lee , H. S. Kim , D. Lee , et al., “Clinical Signs, Duodenal Histopathological Grades, and Serum High‐Mobility Group Box 1 Concentrations in Dogs With Inflammatory Bowel Disease,” Journal of Veterinary Internal Medicine 35 (2021): 2205–2214. 34480505 10.1111/jvim.16258 PMC8478061

[64] A. E. Jergens and R. M. Heilmann , “Canine Chronic Enteropathy‐Current State‐Of‐The‐Art and Emerging Concepts,” Frontiers in Veterinary Science 9 (2022): 923013. 36213409 10.3389/fvets.2022.923013 PMC9534534

[65] M. Gulhane , L. Murray , R. Lourie , et al., “High Fat Diets Induce Colonic Epithelial Cell Stress and Inflammation That Is Reversed by IL‐22,” Scientific Reports 6 (2016): 28990. 27350069 10.1038/srep28990 PMC4924095

[66] R. Kobayasi , E. H. Akamine , A. P. Davel , et al., “Oxidative Stress and Inflammatory Mediators Contribute to Endothelial Dysfunction in High‐Fat Diet‐Induced Obesity in Mice,” Journal of Hypertension 28 (2010): 2111–2119. 20616756 10.1097/HJH.0b013e32833ca68c

[67] M. Yabal , N. Müller , H. Adler , et al., “XIAP Restricts TNF‐ and RIP3‐Dependent Cell Death and Inflammasome Activation,” Cell Reports 7 (2014): 1796–1808. 24882010 10.1016/j.celrep.2014.05.008

[68] A. Jergens , J. Young , D. Moore , et al., “Bcl‐2/Caspase 3 Mucosal Imbalance Favors T Cell Resistance to Apoptosis in Dogs With Inflammatory Bowel Disease,” Veterinary Immunology and Immunopathology 158 (2014): 167–174. 24495616 10.1016/j.vetimm.2014.01.004

[69] A. Di Sabatino , R. Ciccocioppo , S. D'Alò , et al., “Intraepithelial and Lamina Propria Lymphocytes Show Distinct Patterns of Apoptosis Whereas Both Populations Are Active in Fas Based Cytotoxicity in Coeliac Disease,” Gut 49 (2001): 380–386. 11511560 10.1136/gut.49.3.380 PMC1728419

[70] N. Calzadilla , S. M. Comiskey , P. K. Dudeja , S. Saksena , R. K. Gill , and W. A. Alrefai , “Bile Acids as Inflammatory Mediators and Modulators of Intestinal Permeability,” Frontiers in Immunology 13 (2022): 1021924. 36569849 10.3389/fimmu.2022.1021924 PMC9768584

[71] J. B. J. Ward , N. K. Lajczak , O. B. Kelly , et al., “Ursodeoxycholic Acid and Lithocholic Acid Exert Anti‐Inflammatory Actions in the Colon,” American Journal of Physiology. Gastrointestinal and Liver Physiology 312 (2017): G550–g558. 28360029 10.1152/ajpgi.00256.2016

[72] M. Imamura , H. Nakajima , H. Takahashi , H. Yamauchi , and G. I. Seo , “Bile Acid Metabolism, Bacterial Bowel Flora and Intestinal Function Following Ileal Pouch‐Anal Anastomosis in Dogs, With Reference to the Influence of Administration of Ursodeoxycholic Acid,” Tohoku Journal of Experimental Medicine 190 (2000): 103–117. 10770619 10.1620/tjem.190.103

[73] J. Zhang , K. He , L. Cai , et al., “Inhibition of Bile Salt Transport by Drugs Associated With Liver Injury in Primary Hepatocytes From Human, Monkey, Dog, Rat, and Mouse,” Chemico‐Biological Interactions 255 (2016): 45–54. 27000539 10.1016/j.cbi.2016.03.019 PMC5891329

[74] P. A. Dawson and S. J. Karpen , “Intestinal Transport and Metabolism of Bile Acids,” Journal of Lipid Research 56 (2015): 1085–1099. 25210150 10.1194/jlr.R054114 PMC4442867

[75] B. Borgström , G. Lundh , and A. Hofmann , “The Site of Absorption of Conjugated Bile Salts in Man,” Gastroenterology 54 (1968): 781–783. 5653800

[76] S. A. Joyce and C. G. Gahan , “Bile Acid Modifications at the Microbe‐Host Interface: Potential for Nutraceutical and Pharmaceutical Interventions in Host Health,” Annual Review of Food Science and Technology 7 (2016): 313–333. 10.1146/annurev-food-041715-033159 26772409

[77] N. M. Sagar , I. A. Cree , J. A. Covington , and R. P. Arasaradnam , “The Interplay of the Gut Microbiome, Bile Acids, and Volatile Organic Compounds,” Gastroenterology Research and Practice 2015 (2015): 398585. 25821460 10.1155/2015/398585 PMC4363917

[78] H. Duboc , S. Rajca , D. Rainteau , et al., “Connecting Dysbiosis, Bile‐Acid Dysmetabolism and Gut Inflammation in Inflammatory Bowel Diseases,” Gut 62 (2013): 531–539. 22993202 10.1136/gutjnl-2012-302578

[79] R. Comito , E. Porru , N. Interino , et al., “Metabolic Bile Acid Profile Impairments in Dogs Affected by Chronic Inflammatory Enteropathy,” Metabolites 13, no. 9 (2023): 980, 10.3390/metabo13090980. 37755260 PMC10535270

[80] A. B. Blake , B. C. Guard , J. B. Honneffer , J. A. Lidbury , J. M. Steiner , and J. S. Suchodolski , “Altered Microbiota, Fecal Lactate, and Fecal Bile Acids in Dogs With Gastrointestinal Disease,” PLoS One 14 (2019): e0224454. 31671166 10.1371/journal.pone.0224454 PMC6822739

[81] L. K. Stenman , R. Holma , A. Eggert , and R. Korpela , “A Novel Mechanism for Gut Barrier Dysfunction by Dietary Fat: Epithelial Disruption by Hydrophobic Bile Acids,” American Journal of Physiology. Gastrointestinal and Liver Physiology 304 (2013): G227–G234. 23203158 10.1152/ajpgi.00267.2012

[82] S. Devkota , Y. Wang , M. W. Musch , et al., “Dietary‐Fat‐Induced Taurocholic Acid Promotes Pathobiont Expansion and Colitis in Il10−/− Mice,” Nature 487 (2012): 104–108. 22722865 10.1038/nature11225 PMC3393783

[83] T. Phungviwatnikul , A. H. Lee , S. E. Belchik , J. S. Suchodolski , and K. S. Swanson , “Weight Loss and High‐Protein, High‐Fiber Diet Consumption Impact Blood Metabolite Profiles, Body Composition, Voluntary Physical Activity, Fecal Microbiota, and Fecal Metabolites of Adult Dogs,” Journal of Animal Science 100 (2022). 10.1093/jas/skab379 PMC8846339 34967874

[84] S. Wang , R. Martins , M. C. Sullivan , et al., “Diet‐Induced Remission in Chronic Enteropathy Is Associated With Altered Microbial Community Structure and Synthesis of Secondary Bile Acids,” Microbiome 7 (2019): 126. 31472697 10.1186/s40168-019-0740-4 PMC6717631

